# Analysis of the Damping Characteristics of Cylindrical Resonators Influenced by Piezoelectric Electrodes

**DOI:** 10.3390/s17051017

**Published:** 2017-05-04

**Authors:** Jiangkun Sun, Yulie Wu, Xiang Xi, Yongmeng Zhang, Xuezhong Wu

**Affiliations:** College of Mechatronics Engineering and Automation, National University of Defense Technology, Changsha 410073, China; sjknudt@163.com (J.S.); fordada@126.com (X.X.); zymnudt@163.com (Y.Z.)

**Keywords:** damping characteristic, FE model, piezoelectric electrodes, adhesive layer

## Abstract

The cylindrical resonator gyroscope (CRG) is a typical Coriolis vibratory gyroscope whose performance is mostly influenced by the damping characteristic of the cylindrical resonator. However, the tremendous damping influences caused by pasting piezoelectric electrodes on the gyroscope, which degrades the performance to a large extent, have rarely been studied. In this paper, the dynamical model is established to analyze various forms of energy consumption. In addition, a FE COMSOL model is also created to discuss the damping influences of several significant parameters of the adhesive layer and piezoelectric electrodes, respectively, and then explicit influence laws are obtained. Simulation results demonstrate that the adhesive layer has some impact on the damping characteristic, but it not significant. The Q factor decreases about 30.31% in total as a result of pasting piezoelectric electrodes. What is more, it is discovered that piezoelectric electrodes with short length, locations away from the outside edges, proper width and well-chosen thickness are able to reduce the damping influences to a large extent. Afterwards, experiments of testing the Q factor are set up to validate the simulation values.

## 1. Introduction

In recent years, there has been a resurgence of interest in solid-state wave gyroscopes because they have advantages of small size, high operational accuracy, low cost, low power consumption, good shock resistance, and long life. Axisymmetric resonators such as hemispherical shells, cylindrical shells or their evolutionary structures are utilized to measure the angular velocity of a rotating body, which is based on the inertia effect of the standing wave in two vibration modes [1]. As a kind of solid-state wave gyroscope, cylindrical resonant gyros (CRGs) have a relatively simple structure compared with hemispherical resonator gyroscopes which make them less complicated to manufacture [2]. Therefore, CRGs with high performance and low cost have a huge competitive advantage and considerable potential for applications in many fields, such as avionics systems, borehole surveying, missiles, naval equipment, platform stabilization and robots [3]. Hence, many researchers, institutes and companies have shown great interest in cylindrical resonant gyros. Watson Industries (Eau Claire, WI, USA) created vibratory structure gyroscopes with a cupped piezoelectric structure. Similarly, Innalabs Holding (Dublin, Ireland) is researching and producing low cost metallic Coriolis vibratory gyroscopes with excellent performance [4].

Piezoelectric electrodes can be utilized for actuation and detection, because they have advantages of high driving efficiency, fast response, wide frequency range and high precision. Vibratory structures with piezoelectric actuators are broadly applied in vibratory gyroscopes such as tuning fork gyroscopes, beam gyroscopes and cylindrical resonant gyroscopes. The common method to utilize piezoelectric electrodes is by pasting them on the vibratory structure, which will certainly alter the local mass, stiffness and damping characteristic of the resonators. In addition, many studies report that piezoelectric electrodes have relatively large damping characteristics. They are usually used as damping shock absorbers to reduce the vibration of vibratory structures through converting mechanical energy into electrical energy [5]. The gyro drift caused by the asymmetric distribution of mass and stiffness can be eliminated through mechanical trimming and force-rebalance closed-loop control [6], however, it is noteworthy that there is no effective way to control or eliminate completely the negative impacts on the damping characteristics, which are an inherent property of the resonator. What is more, a high accuracy CRG requires the resonator’s damping to be small and have a homogeneous distribution. As a consequence, the damping characteristic of pasting PZTs on a gyro will result in some unexpected errors and limit the development of high-performance gyros. 

At present, many researchers are paying much attention to structure design optimization [7], controlling manufacturing errors [8] and modification of materials [9] of resonators to reduce their damping influences. Nevertheless, the damping characteristic of resonators will still experience significant variations after pasting piezoelectric electrodes. However, these influences on the damping characteristics are always neglected and need to be thoroughly researched. This paper will develop an in-depth study in this topic to reveal how the adhesive layer and the parameters of piezoelectric electrodes affect the damping characteristic as evaluated by the Q factor. Afterwards, useful instructions will be provided for improving piezoelectric electrode pasting procedures and enhancing the gyro performance.

## 2. Gyro Description and Theoretical Consideration

### 2.1. Structure and Principle

**Figure 1:**
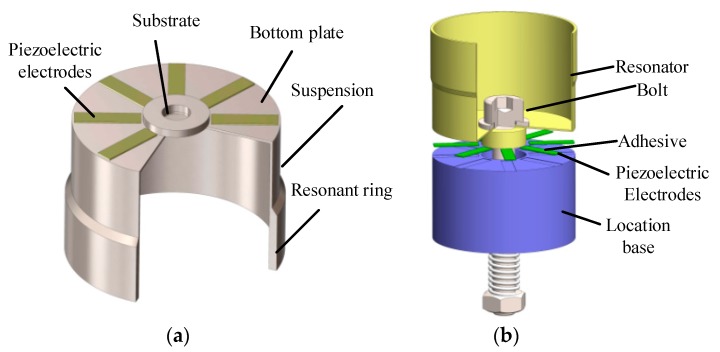
(**a**) Structure of the cylindrical resonator; (**b**) Schematic of the pasting process.

As presented in Figure 1a, the typical structure of a cylindrical shell vibratory gyroscope consists of a rigid substrate, a bottom plate and a cylindrical wall. The bottom plate is connected to the substrate supporting the entire resonator. The resonant ring used for sensing angular velocity is much thicker than the suspension, and it should be precisely manufactured. More importantly, eight piezoelectric electrodes should be pasted on the bottom plate and need to be evenly separated from each other by 45°. The process of pasting piezoelectric electrodes is presented in Figure 1b. Eight grooves on the location base are designed to ensure the precise orientation of the electrodes. In addition, a nut and a spring are used to provide proper strength to the piezoelectric electrodes as they are thin and fragile. After pasting the piezoelectric electrodes, the resonator is dried at 120 °C for about two hours and then the adhesive layer is solidified [10].

**Figure 2:**
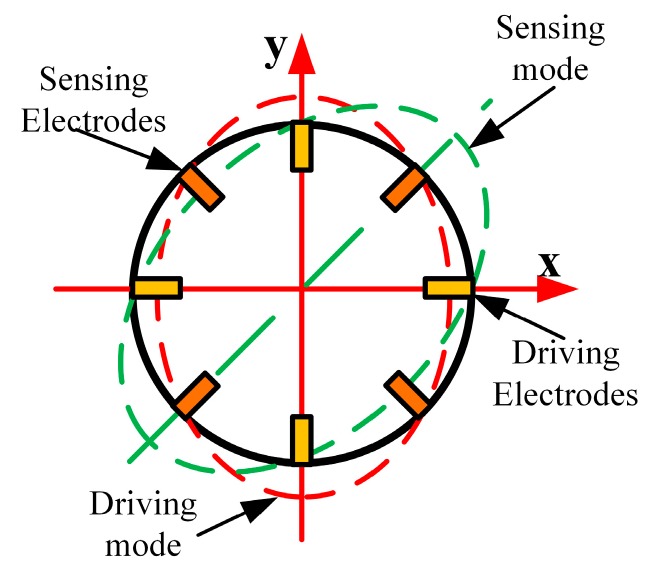
Mode shape of a resonator.

The operational principle of the vibratory cylinder gyroscope is illustrated in Figure 2. Owing to the converse-piezoelectric effect, the resonator is excited into the driving mode through applying a varying voltage on the piezoelectric electrodes pasted on the bottom plate along the *x*-axis. Similarly, the sensing mode will also be excited as a result of the Coriolis force effect when the gyro rotates. Then the angular velocity of the gyro can be obtained by demodulating the electrical signals of sensing electrodes based on the piezoelectric effect. Therefore, precise driving and detection of the piezoelectric electrodes are vital to guarantee the performance of the gyro.

### 2.2. Theoretical Considerations

#### 2.2.1. The Dynamical Model of Piezoelectric Driving and Detection

**Figure 3:**
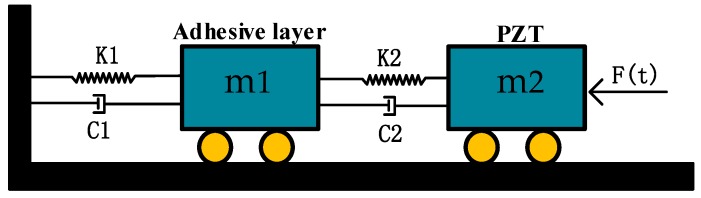
Dynamical model of piezoelectric actuation and detection.

In fact, the piezoelectric driving and detection of a gyro can be regarded as a coupled model of a resonator, an adhesive layer and the PZTs. As is shown in Figure 3, the dynamical model will be helpful for better understanding the vibratory and damping characteristics. Energy loss of the adhesive layer mainly comes from heat generated by friction. Similarly, the damping of piezoelectric electrodes is produced caused by internal dielectric and mechanical losses. To be more specific, the internal dielectric loss is primarily caused by polarization relaxation and leakage loss. Besides, the mechanical loss refers to the segmental energy loss caused by overcoming the internal friction in the vibration of the piezoelectric electrodes [11]. In this dynamical model, the energy loss of these parts is equivalent to two dampers which can also provide the foundation for establishing the FE model. 

The dynamical equation of this model can be expressed as:

(1) $$\left[\begin{array}{cc} m_{1} & 0\\ 0 & m_{2} \end{array}\right]\left\{\begin{array}{c} \ddot{x_{1}}\\ \ddot{x_{2}} \end{array}\right\}+\left[\begin{array}{cc} c_{1}+c_{2} & -c_{2}\\ -c_{2} & c_{2} \end{array}\right]\left\{\begin{array}{c} \dot{x_{1}}\\ \dot{x_{2}} \end{array}\right\}+\left[\begin{array}{cc} k_{1}+k_{2} & -k_{2}\\ -k_{2} & k_{2} \end{array}\right]\left\{\begin{array}{c} x_{1}\\ x_{2} \end{array}\right\}=\left\{\begin{array}{c} 0\\ F\left(t\right) \end{array}\right\}$$

where $m_{1},m_{2},k_{1},k_{2},c_{1},c_{2},x_{1},x_{2}$ are the mass, stiffness, equivalent damping and displacement of the adhesive layer and piezoelectric electrodes, respectively.

The differential equation of the model is converted into Equations (2) and (3) by a Laplace transform:

(2) $$m_{1}s^{2}x_{1}\left(s\right)+\left(c_{1}+c_{2}\right)sx_{1}\left(s\right)-c_{2}sx_{2}\left(s\right)+\left(k_{1}+k_{2}\right)x_{1}\left(s\right)-k_{2}x_{2}\left(s\right)=0$$

(3) $$m_{2}s^{2}x_{2}\left(s\right)-c_{2}sx_{1}\left(s\right)+c_{2}sx_{2}\left(s\right)-k_{2}x_{1}\left(s\right)+k_{2}x_{2}\left(s\right)=F\left(t\right)$$

After substituting s=jω into Equations (2) and (3) and complex numbers operation, the amplitude of the adhesive layer and PZT can be expressed as:

(4) $$x_{1}=\frac{cF\left(t\right)}{ab-c^{2}}$$

(5) $$x_{2}=\frac{bF\left(t\right)}{ab-c^{2}}$$

where $a=k_{2}-m_{2}\omega^{2}+\omega c_{2}j$; $b=k_{1}+k_{2}-m_{1}\omega^{2}+(c_{1}+c_{2})\omega j$; $c=k_{2}+c_{2}\omega j$.

#### 2.2.2. The Loss Factor of Adhesive Layer and PZT

Loss factor is an important parameter to evaluate the damping characteristics of systems and express the energy dissipation of vibration. It is equal to the ratio of energy loss and the maximum energy storage in one cycle [12], so the loss factor η can be defined as follows:

(6) $$\eta =\frac{\Delta W}{W}$$

where ΔW is the energy loss and W is the maximum energy storage in one cycle.

When the adhesive layer is under the excitation of the harmonic force, the displacement phase is always lagging behind the force phase by φ. As a result, the force-displacement hysteresis curve forms an ellipse in one cycle and the area of this ellipse is the energy loss $\Delta W_{1}$ [13].

As a result of the inverse piezoelectric effect, piezoelectric electrodes will generate a mechanical deformation when an alternating voltage $U=U_{0}\sin\left(\omega t\right)$ is applied oon them. In this deformation process, a shear force is transferred to the resonator through the adhesive layer. On the basis of the uniform strain model and the consistent deformation of the adhesive layer and PZT, the shear force $f_{c}$ transferred by the adhesive layer can be described as [14]:

(7) $$f_{c}=\frac{\Lambda bG_{j}}{\gamma^{2}h_{j}}\left[sec\left(\frac{\gamma L}{2}\right)-1\right]\sin\left(\omega t\right)$$

where $\gamma =\sqrt{\frac{\rho hh_{j}\omega^{2}+G_{j}}{c_{11}^{E}hh_{j}}}$ and $G_{j}=\frac{E_{j}}{2\left(1+v_{j}\right)}$, $E_{j},\upsilon_{j},h_{j},\Lambda$ are the elastic modulus, Poisson’s ratio, thickness of the adhesive layer and induced strain, $h,b,L,C_{11}^{E}$ are the thickness, width, length and elastic stiffness of PZT. 

The energy consumption of the adhesive layer and loss factor can be expressed as:

(8) $$\eta_{1}=\frac{\Delta W_{1}}{W_{1}}=\frac{\oint^{\text{​}}f_{c}dx_{1}}{W_{1}}$$

From Equation (8), it can be summarized that the loss factor of the adhesive layer is influenced by the thickness and elastic modulus of the adhesive layer and the dimensional parameters of the PZT.

As for the mechanical loss of piezoelectric electrodes, the energy consumption caused by internal friction under a cycle can be approximately regarded as proportional to the square of the vibratory amplitude [15]:

(9) $$E_{J}=\int_{0}^{\frac{2\pi }{\omega }}\alpha {\left|x_{2}\left(t\right)\right|}^{2}dt$$

where $E_{J}$ is the mechanical loss, and α is the scale factor.

Similarly, the internal dielectric loss can be seen as the electric energy generated by a PZT with a static load. When the piezoelectric electrodes bear a load F, the place of x on the PZT will produce an electricity charge which can be described as:

(10) $$Q=\int_{z_{p}-t_{p}}^{z_{p}}d_{31}\frac{Fx}{I_{z}}bzdz=\int_{z_{p}-t_{p}}^{z_{p}}d_{31}\frac{1}{I_{z}}bzdz\cdot Fx=MFx$$

where $M=\int_{z_{p}-t_{p}}^{z_{p}}d_{31}\frac{1}{I_{z}}bzdz$, $z_{p}$ is the distance between the top surface and the neutral axis of PZT, $I_{Z}$ is the moment of inertia, x is the distance from the edge of PZT along the length direction and $d_{31}$ is piezoelectric constant. 

The static capacitance of a piezoelectric electrode is approximately regarded as proportional to its area. The total capacitance would be bLC, assuming the capacitance of the unit area is C. When the piezoelectric electrode bears the force $F=F_{0}\sin\left(\omega t\right)$, its electric energy loss $E_{D}$ can be expressed as:

(11) $$E_{D}=\int_{0}^{\frac{2\pi }{\omega }}\int_{0}^{l}UQdxdt=\int_{0}^{\frac{2\pi }{\omega }}\int_{0}^{l}\frac{{\left[Mx\cdot F\sin\left(\omega t\right)\right]}^{2}}{bLC}dxdt$$

So the loss factor of piezoelectric electrodes is denoted as:

(12) $$\eta_{2}=\frac{\Delta W_{2}}{W_{2}}=\frac{E_{J}+E_{D}}{W_{2}}$$

where $\Delta W_{2}$ is the energy loss and $W_{2}$ is the maximum energy storage in one cycle of piezoelectric electrodes’ vibration. As Equation (12) suggests, the loss factor of piezoelectric electrodes is affected by the dimensional parameters of the piezoelectric electrodes.

## 3. Modeling and Analysis

Piezoelectric electrodes are pasted on the bottom of resonator with an adhesive layer, forming a PZT-adhesive layer-resonator coupled model. However, the model’s damping characteristic is related to a complicated dynamic theory and numerical solutions are hard to obtain. As a result, the finite element model (FEM) method is applied to analyze the damping characteristic of the resonator to get the analytical solutions in this study.

### 3.1. Finite Element Modeling of the Resonator

The damping simulation types of COMSOL consist of Rayleigh damping, isotropy loss factor and so on. There is no precise way to simulate the damping characteristic of the coupled model on account of its complexity. On the basis of the dynamical model above, the loss factor is selected as the damping parameter to represent the damping characteristics of the three sections which will be helpful for simplifying the problem and improving the efficiency of the simulation. Several significant material parameters of nickel alloy (Ni_42_CrTiAl), adhesive layer and PZT (PZT-5H) are listed in Table 1 to meet the demands of the damping simulation. The loss factors of nickel alloy and PZT are obtained from experimental measurements and the adhesive layer’s loss factor comes from the handbook [16]. 

**Figure 4:**
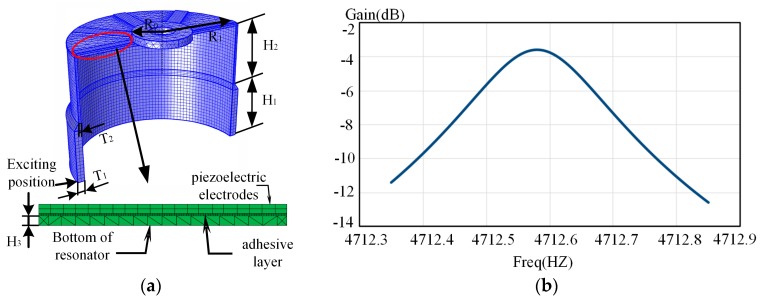
The simulation model and result: (**a**) Finite element model; (**b**) Simulation result.

Some geometry parameters listed in Table 2 are used to build the model. The meshed model of the resonator is constructed by the COMSOL software, as illustrated in Figure 4. In order to obtain a more accurate result, the piezoelectric electrodes, the adhesive layer and resonator are divided into a homogeneous cuboid and tetrahedron to form a suitable finite element model. Then, the Q factor can be calculated through frequency-domain analysis around the resonant frequency. 

### 3.2. Simulation Analysis of Adhesive Layer

The adhesive layer serving as the connector between the piezoelectric electrodes and resonator may have an influence on the damping characteristic of the gyro. In order to establish the piezoelectric electrodes’ effects more clearly, the influence of the adhesive layer must be considered first. Therefore, this paper mainly focuses on several important factors of the adhesive layer such as thickness and elastic modulus, which will provide effective suggestions concerning the pasting process and reduce its negative effects.

#### 3.2.1. Influences of Adhesive Layer’s Thickness

**Figure 5:**
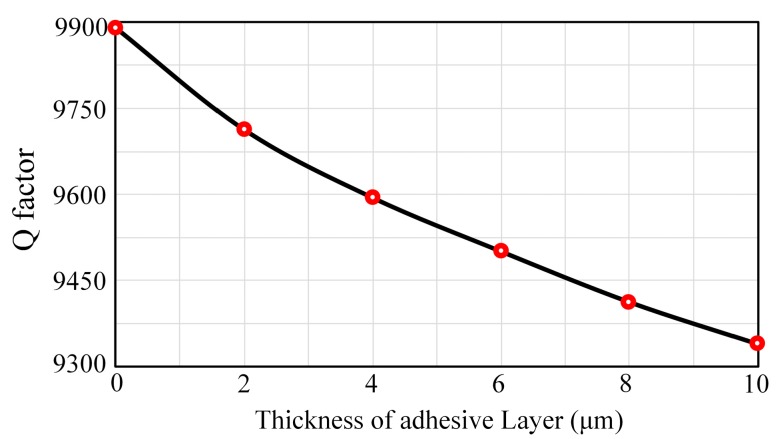
Q factors for different thicknesses.

In this simulation process, the adhesive layer’s thickness varies from 0 μm (rigid connection) to 10 μm. Other material properties should remain unchanged and the elastic modulus is set to be 5 GPa. As shown in Figure 5, the Q factor declines about 5.68% with the increase of thickness. The energy transfer efficiency will decrease when the adhesive layer’s thickness increases because there will be more energy consumption in the adhesive layer during the vibration, so the thinner the adhesive layer, the less impact it will have on the damping characteristics. However, controlling its thickness under 5 μm is really hard and it is also difficult to ensure the uniformity of the pasting adhesive layer. As a result, the thickness of the adhesive layer should be controlled around 5 μm to reduce its effect on the damping characteristics and guarantee its uniformity at the same time.

#### 3.2.2. Influences of Adhesive Layer’s Elastic Modulus

**Figure 6:**
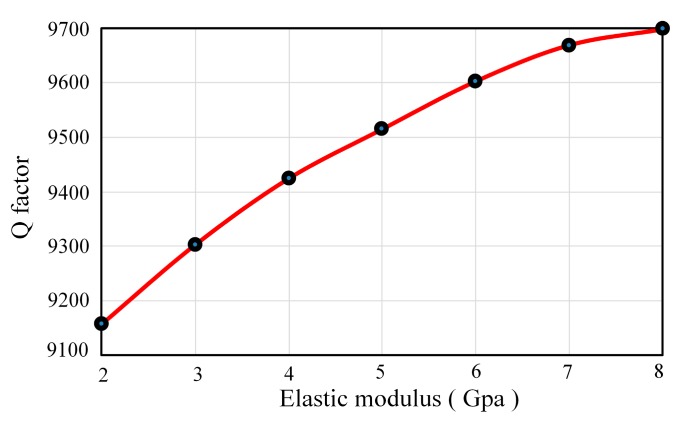
Q factor under different elastic modulus.

When the adhesive layer congeals and solidifies, its elastic modulus depends on the material properties. Moreover, its elastic modulus also has an influence on the damping characteristics. At present, a typical adhesive layer consists of an epoxy resin adhesive and a conductive adhesive. In order to figure out the effects of elastic modulus, it is changed from 2 GPa to 8 GPa while the other properties remain the same (thickness is 5 μm). As Figure 6 shows, when the elastic modulus of the adhesive layer increases, the Q factor increases about 6.02% and the growth rate declines gradually. It will be better to select an adhesive layer with a large elastic modulus to reduce the damping influences.

From the analysis of results given above, it can be summarized that the adhesive layer between piezoelectric electrodes and resonator has some influence on the damping characteristics to a certain degree. Therefore, thin thickness and large elastic modulus of the adhesive layer will decrease the influence on damping characteristic and enhance the gyro performance. 

### 3.3. Simulation Analysis of Piezoelectric Electrodes’ Parameters

There is no doubt that pasting piezoelectric electrodes will alter the damping characteristics of the resonator through the stress hysteresis effect and dynamical loss, so it is necessary to perform a thorough inquiry about the degree of impact of several piezoelectric electrode parameters such as length, width, thickness and location, etc. In addition, the dimensions of the piezoelectric electrodes currently used on the CRG are 8 mm (length) × 2 mm (width) × 0.2 mm (thickness). Hence, the variation ranges of length, width and thickness are based on 8 mm, 2 mm and 0.2 mm, respectively.

#### 3.3.1. Influences of Piezoelectric Electrodes’ Length and Width

The length and width of piezoelectric electrodes constitute the piezoelectric plane which mainly determines its driving and detection ability. In the process of this simulation, the length L of two pairs of PZTs was altered from 8 mm to 0 mm (without PZT) along the shortened direction and its inner edges are always next to the substrate. Piezoelectric electrodes may interfere with each other if the width B is too large, so the width B was changed from 0 mm to 4 mm while the other parameters remain the same.

Overall, when two pairs of piezoelectric electrodes (8 mm × 2 mm × 0.2 mm) are pasted on the bottom of the resonator, the damping characteristics vary considerably with the Q factor decreasing about 30.31% from 13663.52 to 9522.42. Therefore, it is not difficult to notice that the piezoelectric electrodes exert a tremendous influence on the damping characteristics of resonators.

**Figure 7:**
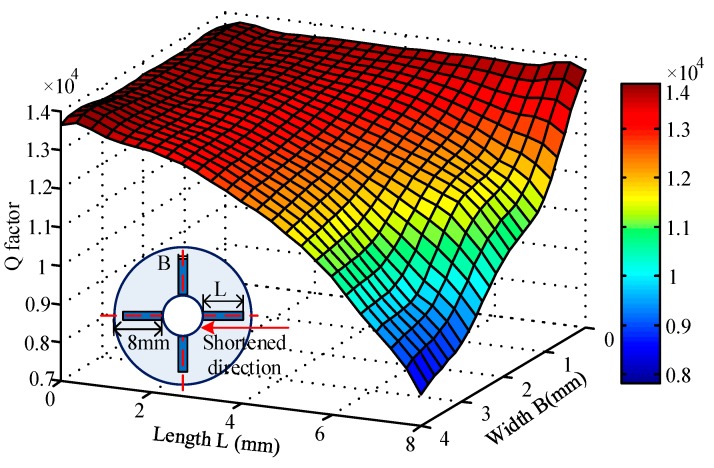
Q factor distribution with different lengths and widths of the piezoelectric electrodes.

As shown in Figure 7, the Q factor increases continuously with the decreasing length of the PZT while the growth rate declines gradually. What is more, it can be briefly summarized that the outside edges of the piezoelectric electrodes have a greater impact on the damping characteristics than the inner edges. In addition, the influences of the outside edges increase gradually as the width broadens. As for width, it is obvious that the Q factor goes up quickly with decreasing width and its upward trend ascends step by step. Moreover, the damping influence of the width’s modification is becoming smaller along the length shortened direction.

**Figure 8:**
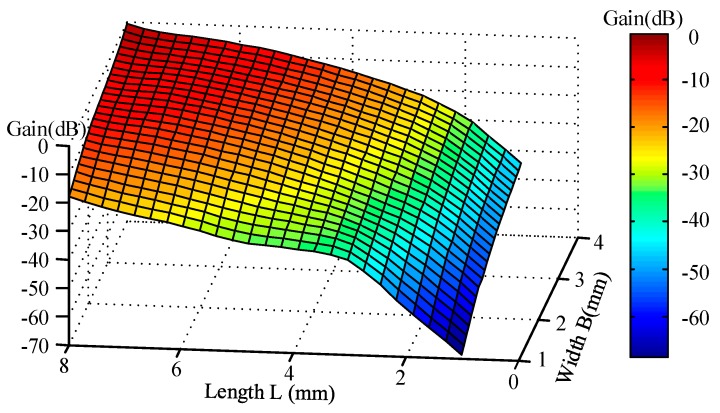
Gain distribution with different lengths and widths of the piezoelectric electrodes.

At the same time, it must not be ignored that the gain of piezoelectric actuation also experiences great changes. As is shown in Figure 8, when the width and length are rising, the gyro’s gain increases gradually with a slowing down rate of ascent. Similarly, it is easy to notice that the gain of variation is mainly influenced by length direction.

#### 3.3.2. Influences of the Piezoelectric Electrodes’ Thickness

As for the dimensions of the piezoelectric electrodes, the thickness, which is usually less than 0.4 mm, is really tiny compared to the length and width. However, it is the direction of polarization which is really significant for the piezoelectric effect. Therefore, it is necessary to consider its influence on the damping characteristics. 

**Figure 9:**
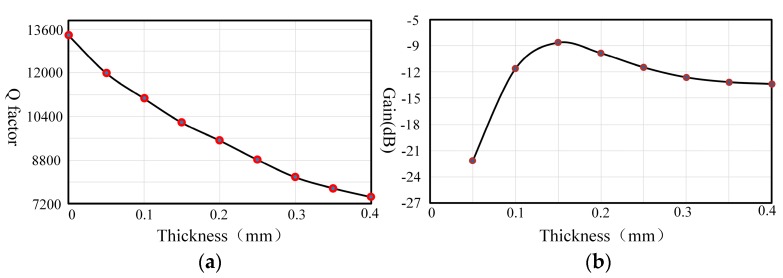
Variation due to the piezoelectric electrodes’ thickness: (**a**) Q factor variation; (**b**) The gain variation of piezoelectric driving.

As shown in Figure 9, the Q factor decreases approximately linearly and changes about 44.12% with the increasing thickness. However, the gyro gain experiences the process of increasing rapidly when the thickness is less than 0.15 mm. After that, it decreases gradually and its trend becomes slower, so piezoelectric electrodes have an optimum thickness for the gyro gain. The optimum thickness is around 0.15 mm, which is about half of the resonator’s bottom thickness (0.3 mm). If the thickness is selected to be too thin, the driving energy of the piezoelectric electrodes is reduced due to piezoelectric material’s characteristics, which will do great harm to the piezoelectric actuation gain. On the contrary, the piezoelectric electrodes will limit the vibration of the resonator when they are too thick. This conclusion is consistent with the results of the reference [17]. Hence, it is paramount to ensure the gyro’s gain through choosing a thickness around 0.15 mm.

#### 3.3.3. Influences of the Piezoelectric Electrodes’ Location

**Figure 10:**
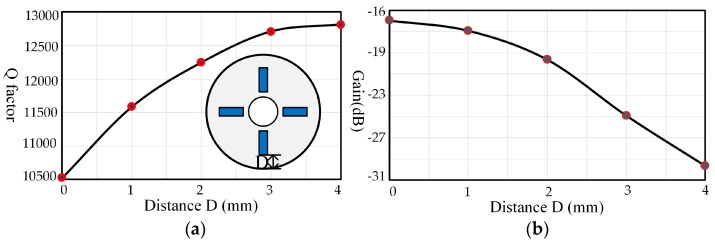
Variation due to piezoelectric electrodes’ location: (**a**) Q factor variation; (**b**) The gain variation of piezoelectric driving.

The locations of the piezoelectric electrodes are significant parameters for the pasting process, which may result in various damping effects. In order to investigate the influences of location, two pairs of piezoelectric electrodes with medium size (4 mm × 2 mm × 0.2 mm) are pasted on the bottom and they are moved from the outside edges to the inner edges. As Figure 10 shows, the Q factor presents a rising tendency, varying about 21.72% as the distance D increases, but this uptrend is becoming slower. On the contrary, the gain decreases gradually with an increasing trend.

## 4. Experiments and Discussion

### 4.1. Experimental Setup

**Figure 11:**
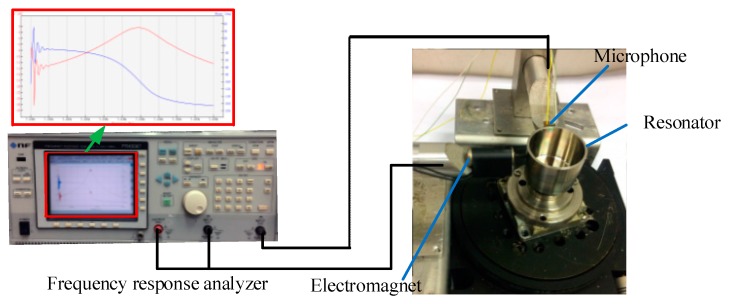
Experimental system.

An experimental system is implemented to measure the Q factor by using the acoustic method to verify the simulation results discussed above. In this experiment, the resonator and piezoelectric electrodes’ materials are in accordance with the simulation. The adhesive layer adopts a conductive adhesive and its elastic modulus is around 5 Gpa. As shown in Figure 11, the experimental testing equipment consists of a microphone for gathering vibration signals, electromagnet for driving the resonator and a frequency response analyzer (NF FRA5087, Yokohama, Japan) for signal generation and analysis. 

The Q factor is measured by frequency response analysis and the procedure of this experiment is as follows: firstly, the frequency response analyzer generates sine sweep signals with frequency around the resonant frequency f. In order to make the measurement results more accurate, the sweep range of the FRA is configured from f−0.5 Hz to f+0.5 Hz and the resolution of the sweeping signals is 0.001 Hz. Afterwards, the sweeping frequency signals are transferred to the electromagnet to excite the resonator. Simultaneously, the vibration signals of the resonator are gathered by the microphone and transmitted back to the FRA. Finally, the Q factor is calculated through vibration signal analysis.

However, there are some issues that need to be addressed to make the experiment more accurate. Firstly, the frequency split of the resonator with piezoelectric electrodes should be under 0.1 Hz. Otherwise it will have a negative impact on the measurement accuracy. Secondly, when the experiment of altering piezoelectric electrodes’ length and width is conducted, the results will be more accurate if pasting repeatedly piezoelectric electrodes of different dimensions can be avoided. In this experiment, piezoelectric electrodes are only pasted once with the maximum dimension and then removed gradually by laser cutting to change their length and width.

### 4.2. Results and Discussion

**Figure 12:**
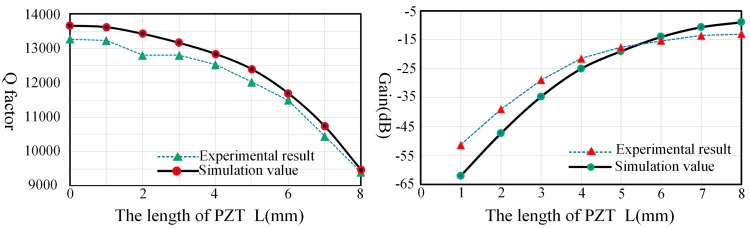
Q factor (**Left**) and gain (**Right**) comparison of experiment and simulation under different PZT lengths.

Figure 12 describes the Q factor and gain comparison of the experiment and simulation under different lengths and they are consistent with each other. Thus, we can conclude that the outside edges of piezoelectric electrodes will generate greater damping effects than the inner edges. Hence, the piezoelectric electrodes’ damping characteristic can be reduced by removing the outside edges of piezoelectric electrodes without losing too much gain. 

**Figure 13:**
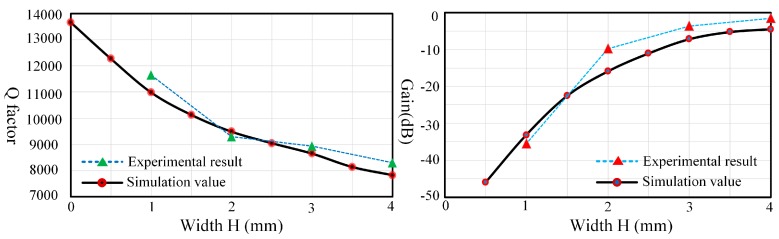
Q factor (**Left**) and gain (**Right**) comparison of experiment and simulation under different PZT widths.

As shown in Figure 13, the experimental results and simulation values display consistent trends. The results show that the width has a significant impact on the damping characteristics and there is a declining trend in the degree impact as the width is broadened. It is critically important to choose a proper width to reduce its impact and guarantee the driving gain at the same time. 

**Figure 14:**
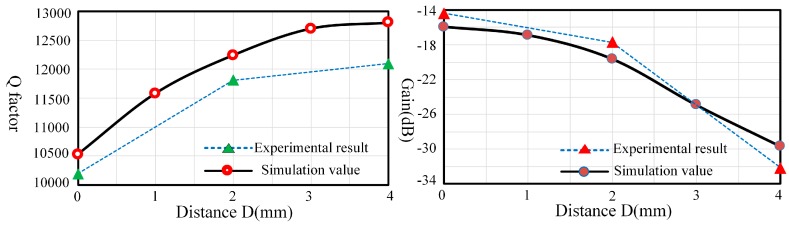
Q factor (**Left**) and gain (**Right**) comparison of experiment and simulation under different locations.

In the case of locations, there is a certain error between experimental results and simulation values because there is no way to avoid pasting piezoelectric electrodes repeatedly to figure out the influences. However, the trends of experimental results and simulation values are similar. As shown in Figure 14, the damping influences of the outside locations take up a great proportion. Therefore, locations away from the outside edges would be a good choice to reduce the impacts of piezoelectric electrodes.

## 5. Conclusions

In this paper, the damping characteristics of cylindrical resonators influenced by piezoelectric electrodes have been investigated in detail. A dynamical model and FE model are established to analyze the damping characteristics. Then the influencing laws of some parameters of the adhesive layer and piezoelectric electrodes are obtained from the simulation results. Afterwards, a series of experiments are implemented to validate the simulation results, showing that the simulation values and experimental results are in accord with each other.

Above all, the adhesive layer between the piezoelectric electrodes and the resonator has some impact on the damping characteristics to a certain extent, but not significantly. Furthermore, an adhesive layer with thin thickness and proper elastic modulus should be selected to reduce its damping influence.

In comparison, piezoelectric electrodes have prominent impacts on the damping characteristics of the resonator and result in a Q factor loss of about 30.31% in total. The width of the piezoelectric electrodes has a significant effect on the damping characteristics and it is critically important to choose a reasonably short width to reduce its influence and guarantee the piezoelectric driving gain at the same time. As for the thickness, it should be a primary selection to make it half of the thickness of the resonator’s bottom to reduce its damping influences and ensure the gyro gain. Additionally, the outside edges have much greater influences on the damping characteristics than the inner edges, so shortening the length of the outside edges or moving the locations away from the outside edges will be a very effective way to reduce its influences on the damping characteristics.

Therefore, a multi-parameter optimization of PZT dimensions can be carried out to decrease the influence and improve the performance of gyros based on the influencing laws of length, width and locations presented in this paper.

## Figures and Tables

**Table 1:** Material parameters of the coupled model.

Name	Density (kg/m^3^)	Elastic Modulus (GPa)	Poisson’s Ratio	Loss Factor
Nickel alloy	8050	206	0.3	$7.3\times 10^{-5}$
Adhesive layer	1760	-	0.38	$6.7\times 10^{-1}$
PZT	7500	60	0.36	$5.2\times 10^{-2}$

**Table 2:** Structural parameters of the resonator.

Parameter	Value (mm)
Height of resonant ring H_1_	8
Height of suspension ring H_2_	10
Radius of substrate R_0_	4
Internal diameter of resonator R_1_	12
Thickness of resonant ring T_1_	1
Thickness of suspension ring T_2_	0.3
Thickness of bottom H_3_	0.3
